# Extracellular matrix-related genes play an important role in the progression of NMIBC to MIBC: a bioinformatics analysis study

**DOI:** 10.1042/BSR20194192

**Published:** 2020-05-26

**Authors:** Heng Zhang, Gang Shan, Jukun Song, Ye Tian, Ling-Yue An, Yong Ban, Guang-Heng Luo

**Affiliations:** 1 Medical School of Guizhou University, Jiaxiu South road, Guiyang, Guizhou Province, P.R. China; 2 Department of Urology Surgery, Guizhou Province People’s Hospital, No. 83 East Zhongshan Road, Guiyang, Guizhou Province, P.R. China; 3 Department of Oral and Maxillofacial Surgery, Guizhou Provincial People’s Hospital, No. 83 East Zhongshan Road, Guiyang, Guizhou Province, P.R. China; 4 Guangzhou Medical University, New town, Panyu district, Guangzhou Province, P.R. China

**Keywords:** Bladder cancer, Extracellular matrix, GEO

## Abstract

Bladder cancer is the 11th most common cancer in the world. Bladder cancer can be roughly divided into muscle invasive bladder cancer (MIBC) and non-muscle invasive bladder cancer (NMIBC). The aim of the present study was to identify the key genes and pathways associated with the progression of NMIBC to MIBC and to further analyze its molecular mechanism and prognostic significance. We analyzed microarray data of NMIBC and MIBC gene expression datasets (GSE31684) listed in the Gene Expression Omnibus (GEO) database. After the dataset was analyzed using R software, differentially expressed genes (DEGs) of NMIBC and MIBC were identified. These DEGs were analyzed using Gene Ontology (GO) enrichment, KOBAS-Kyoto Encyclopedia of Genes and Genomes (KEGG) pathway analysis, and protein–protein interaction (PPI) analysis. The effect of these hub genes on the survival of bladder cancer patients was analyzed in The Cancer Genome Atlas (TCGA) database. A total of 389 DEGs were obtained, of which 270 were up-regulated and 119 down-regulated. GO and KEGG pathway enrichment analysis revealed that DEGs were mainly involved in the pathway of protein digestion and absorption, extracellular matrix (ECM) receiver interaction, phantom, toll-like receptor (TLR) signaling pathway, focal adhesion, NF-κB signaling pathway, PI3K/Akt signaling pathway, and other signaling pathways. Top five hub genes COL1A2, COL3A1, COL5A1, POSTN, and COL12A1 may be involved in the development of MIBC. These results may provide us with a further understanding of the occurrence and development of MIBC, as well as new targets for the diagnosis and treatment of MIBC in the future.

## Introduction

Bladder cancer is the 11th most common cancer in the world, causing 3.2 deaths per 100000 males and 0.9 deaths per 100000 females every year [1]. Bladder cancer can be roughly divided into muscle invasive bladder cancer (MIBC) and non-muscle invasive bladder cancer (NMIBC) depending on whether it infiltrates the bladder muscle layer or not. Approximately 75% of bladder cancer patients have NMIBC, which has a better prognosis than MIBC [2]. The treatment for NMIBC is a transurethral resection of the bladder tumor (TURBT) plus bladder drug perfusion, but the first choice for MIBC is radical cystectomy (RC) [3]. It is apparent that muscle invasion in bladder cancer patients has an important impact on determining the appropriate treatment plan and prognosis. At present, there are some theories about the molecular mechanisms involved in NMIBC progression, including oncogene activation [4], immune regulation [5], and extracellular matrix (ECM) alterations [6,7].

In recent years, gene expression chip technology, which has been widely used in oncology research, has been used to explore the gene expression profile of tumor cells more completely, and this biological information is of great significance for the diagnosis, treatment, and prognosis of the tumor [8]. With the wide application of gene expression arrays, more and more chip data are being published in public databases, and the data integration and mining of these public databases can help us understand the changes occurring in a tumor at a deeper level.

In the present paper, a microarray dataset of gene expression profiles (GSE31684) was accessed. After the dataset was analyzed using R software, differentially expressed genes (DEGs) of NMIBC and MIBC were identified. These DEGs were analyzed using Gene Ontology (GO) enrichment, KOBAS-Kyoto Encyclopedia of Genes and Genomes (KEGG) pathway analysis, and protein–protein interaction (PPI) analysis. The effect of these hub genes on the survival of bladder cancer patients was analyzed in The Cancer Genome Atlas (TCGA) database. Based on the above analysis, we have obtained the DEGs related to the occurrence and development of bladder cancer muscle invasion. In the present paper, we discuss the cell biological functions, biological signal pathways, and the interaction networks of the encoded proteins involved in these DEGs, so as to provide new ideas and possible diagnostic and treatment targets of bioinformatics-related changes involved in the occurrence and development of bladder cancer.

## Materials and methods

### Microarray data

NMIBC and MIBC gene expression datasets GSE31684 were downloaded from Gene Expression Omnibus (GEO, https://www.ncbi.nlm.nih.gov/geo/). GSE31684 has a total of 15 NMIBC samples and 78 MIBC samples (platform: gpl570, [hg-u133_plus_] Affymetrix human genome U133 Plus 2.0 array) [9]. The clinicopathologic characteristics are summarized in Supplementary Table S1.

### Data processing

The downloaded dataset was analyzed and searched for DEGs using R software 3.4.0 (https://www.r-project.org/). The ID corresponding to the probe name was converted into the gene symbol and saved in the txt file. The limma package for bioconductor (http://www.bioconductor.org/) was used to analyze the progressive gene differential expression between NMIBC and MIBC. The conditions were set as *P*<0.05, log fold change (FC) > 1. TXT text results were retained for subsequent analysis.

### Functional and pathway enrichment analysis

The online gene annotation, visualization, and integrated discovery bioinformation resource database (DAVID, Database for Annotation, Visualization, and Integrated Discovery; https://david.ncifcrf.gov/) released in 2003 contains a complete high levels of evidence biological database and a comprehensive analysis tools for systematic and integrative annotation and enrichment analysis that can be used to reveal biological meaning related to large gene lists. We used DAVID to analyze the function and pathway enrichment of the obtained DEGs. GO enrichment analysis was carried out on the webpage using DAVID’s online tools, including biological process (BP), molecular function (MF), and cell component (CC). In addition, GO full analysis was carried out using Cytoscape’s bioapplication program. KEGG path analysis was also carried out on the web using DAVID’s online tools. All the above results were displayed with *P*<0.05.

### PPI network generation and module analysis

Using the STRING database (http://string-db.org/) for PPI network analysis, each point represents a gene, protein, or molecule, and the connection between points represents the interaction between the two molecules. The results in this database are obtained from experimental data, databases, text mining, and predictive bioinformatics data. This type of analysis can show the molecular action and pathway relationship of the DEGs involved in progression from NMIBC to MIBC. However, the gene represented by the central point may be the key protein or candidate gene with important physiological regulatory function. The results of PPI network analysis of hub genes were analyzed using the cytoHubba application of Cytoscape and sorted according to the scores.

### Validation and overall survival analysis of the target genes in TCGA

TCGA database (https://portal.gdc.cancer.gov/) was used to verify the expression of the top five hub genes, and Kaplan–Meier curves were used to compare the impact of these expressed genes on overall survival.

## Results

### Microarray data information and identification of DEGs in MIBC

**Figure 1:**
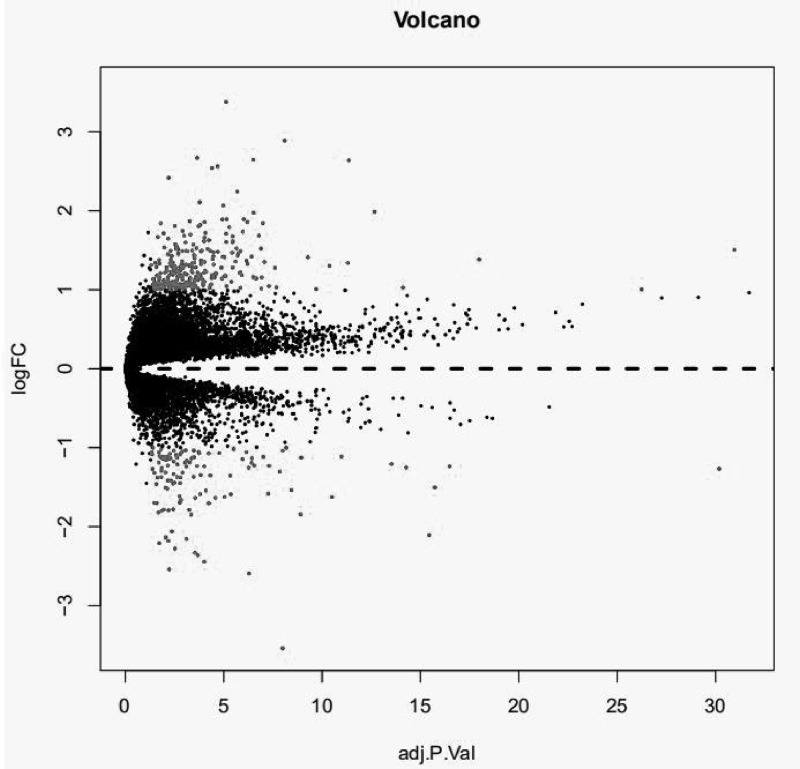
Differential expression of data between two sets of samples The red points represent up-regulated and down-regulated genes screened on the basis of |FC| > 2.0 and a corrected *P*-value of <0.05. The black points represent genes with no significant difference.

**Figure 2:**
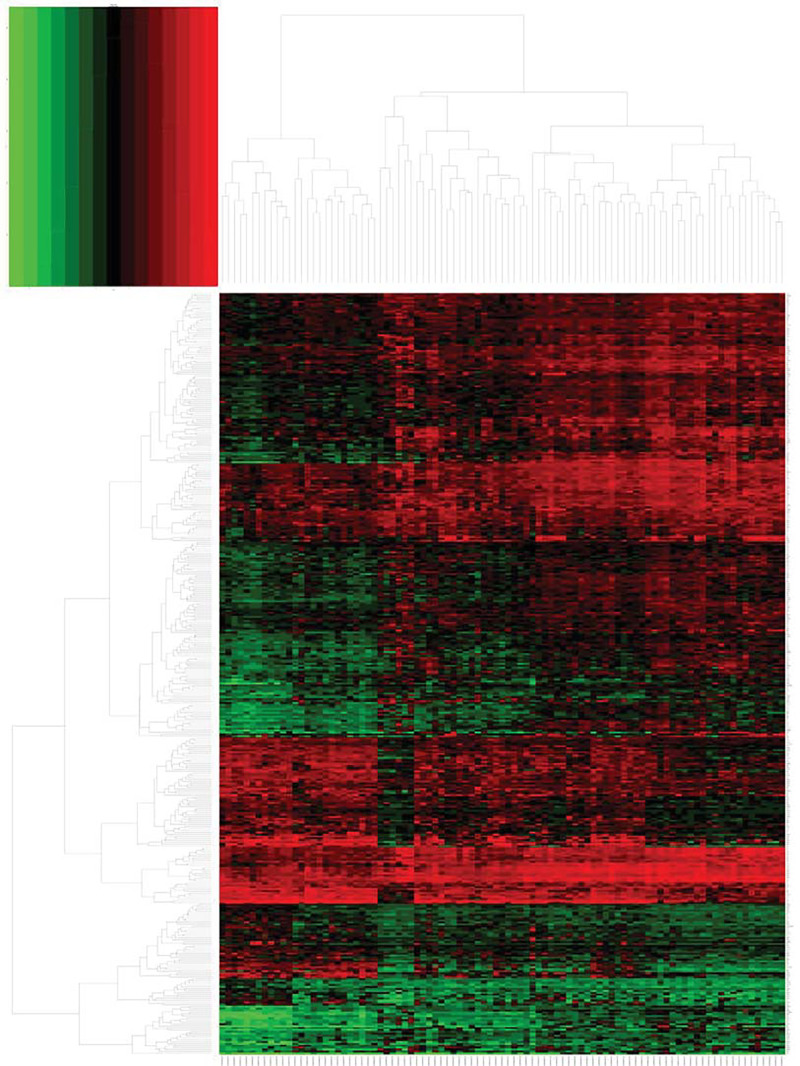
Hierarchical clustering heatmap of DEGs screened on the basis of |FC| > 2.0 and a corrected *P*-value <0.05

The dataset GSE31684 of NMIBC and MIBC tissue gene expression profiles was normalized using the Affy package Robust Multi-array Average (RMA) method. After difference analysis of the GSE31684 dataset using the limma package, a total of 389 DEGs were obtained; the difference expression volcano map of two different tissue samples in the dataset is shown in Figure 1, and the heat map is shown in Figure 2. Among them, 270 genes were up-regulated and 119 were down-regulated (Table 1).

**Table 1:** Screening DEGs by integrated microarray

DEGs	Gene names
**Up-regulated**	FHL1 LIPG LOC100128239 IL6 GEM FAM129A MILR1 KDELC1 NID2 ARSJ PLAU TRIM59 ANP32E CCDC109B TNFSF13B OAT TSPAN4 PSPH H2BFS TMEM158 CXorf21 FBXO17 DUSP14 COX7B2 GPR65 MT1H CD14 HLA-DMB GLIPR1 EMILIN1 TAGLN SLIT2 SYNM KIAA0922 SHOX2 CDKN3 SLFN11 TRIP13 KLHL13 COL12A1 MFAP4 SCG5 EVI2A COL6A2 KCND2 RACGAP1 CDC25B ISLR DACT1 C1QC CAP2 MUM1L1 C1R UBE2E2 PRKCDBP LAP3 MMP11 CDH11 C8orf88 FERMT2 EMP3 SERPING1 FXYD6 CHST11 TIMP1 MAMDC2 PDGFC CTSK CYBRD1 CTSV TUBB6 SULF2 NEXN C5orf34 DCLK1 PRTFDC1 PCOLCE PNMA1 SLC7A7 CKS2 BCAT1 COL15A1 OGN KIAA0895 EPB41L3 PTRF CDC20 PBK SHCBP1 CLIC4 PDGFRL LINC01279 SERPINA3 CNN1 JAZF1 SUGCT MRGPRF DPYSL3 HMCN1 FIBIN CEP55 COL8A2 CAV1 PALLD CYP1B1 KIAA1598 TPST1 AHNAK2 CLEC2B MS4A4A OGFRL1 MN1 CNN3 NXN EVI2B ASB5 MT2A ACTG2 TRPS1 GBP5 GPC6 TREM1 FBN1 OSR2 SYNC CD163 IFIT1 SECTM1 PLAGL1 FBLN2 PCDHB5 NR3C1 MT1HL1 CACNA2D1 PLA2G7 SOSTDC1 STEAP1 PCOLCE2 MGC24103 PLSCR4 CXCL12 SGPP1 TMEM200A MT1E C5AR1 ASPN RNASE6 KATNAL1 AAED1 ALDH2 BCL2A1 OLFML3 TNFAIP6 DEPDC7 DSCR8 CRISPLD1 PEG10 ARSI IGF2BP3 CCL4 MT1X NAP1L3 PI3 TIMP2 DEPDC1B GLT8D2 ZNF521 C3orf80 CXCL10 EPYC ANLN KIAA1524 MGP CXCL9 CCL8 MLLT11 CFL2 TUBA1A RAB23 CD109 CHI3L1 FKBP10 FN1 C1QB FCER1G HIST1H3G ITGAM NUF2 CHRDL2 LOC100132891 CENPW LINC01094 PPAPDC1A CCL19 GXYLT2 KIF18A INHBA ADAMDEC1 IFI6 TGFBI HSD17B6 ANXA5 FLNA COL16A1 COLEC12 COL5A1 RARRES2 OLR1 OXCT1 THBS2 SERPINF1 DSC2 IL18 COL6A3 GPR1 SRPX GAS1 LY96 ROR2 DEGS1 COL1A2 S100A8 FNDC1 COL5A2 C1S VCAN LGALS1 LOX CLMP TUBB2A CPVL COL3A1 TMEM45A PRRX1 CXCL11 HS3ST3A1 MMP9 FCGR3B MMP3 EFEMP1 KRT6B AEBP1 SLC16A1 SULF1 SACS MFAP5 SFRP4 PLN ANXA3 GPX8 FAP ZFPM2 PCP4 MIR100HG SGCE SELM EGR2 POSTN COMP GPNMB KRT6A COL10A1 CTHRC1 PCDHB2 COL11A1 SFRP2 CCL18 GREM1
**Down-regulated**	SLC14A1 BTBD16 SNX31 CYP4B1 HSD17B2 ANXA10 CLCA4 SPINK1 DHRS2 TOX3 PSCA CYP3A7-CYP3AP1 RNF128 MAL TRIM31 LOC102659288 S100P ADIRF ARL14 HPGD CYP4F8 GPX2 UPK1A FMO9P UCA1 UPK2 DUOX2 VSIG2 SIDT1 CYP3A5 ID1 TFF2 CYP4F3 AGAP11 FAM174B FER1L4 CYP4X1 FAM110C ZNF486 C10orf99 CEL ELF3 TFF1 PPARG C8orf4 LCN2 TMEM45B CRH PTPRR LINC00967 ENTPD3 ZNF432 BCAS1 GGT6 GATA3 FOXA1 CDC42EP5 GSTA1 TMPRSS4 FOXQ1 CYP3A43 AGR2 TMEM139 LRP5L ST3GAL5 BMP5 SLC20A1 GPR110 CAPN5 FBP1 CACNA1D CAPNS2 EPS8L3 TMPRSS2 MYCL GRHL3 SEMA6A SYT8 FOSB HMGCS2 DENND2D MAOA RAPGEFL1 MPPED2 AZGP1 GAREM LGALS4 FOS ZSCAN4 PP14571 PPFIBP2 SLC44A3 EMX2 PADI3 FAM162B KLF5 SLC38A11 BHMT BPIFB1 CYP2J2 PLAT EPHB6 TMEM178A SCIN LOC100287525 AQP3 TNFRSF21 CYP4F11 EIF3C SIGLEC15 SORL1 LOC286272 CAMK2N1 TRAK1 GRAMD1C RASSF6 CYP4F12 CXCL17 ZNF440

### GO term enrichment analysis of DEGs

**Figure 3:**
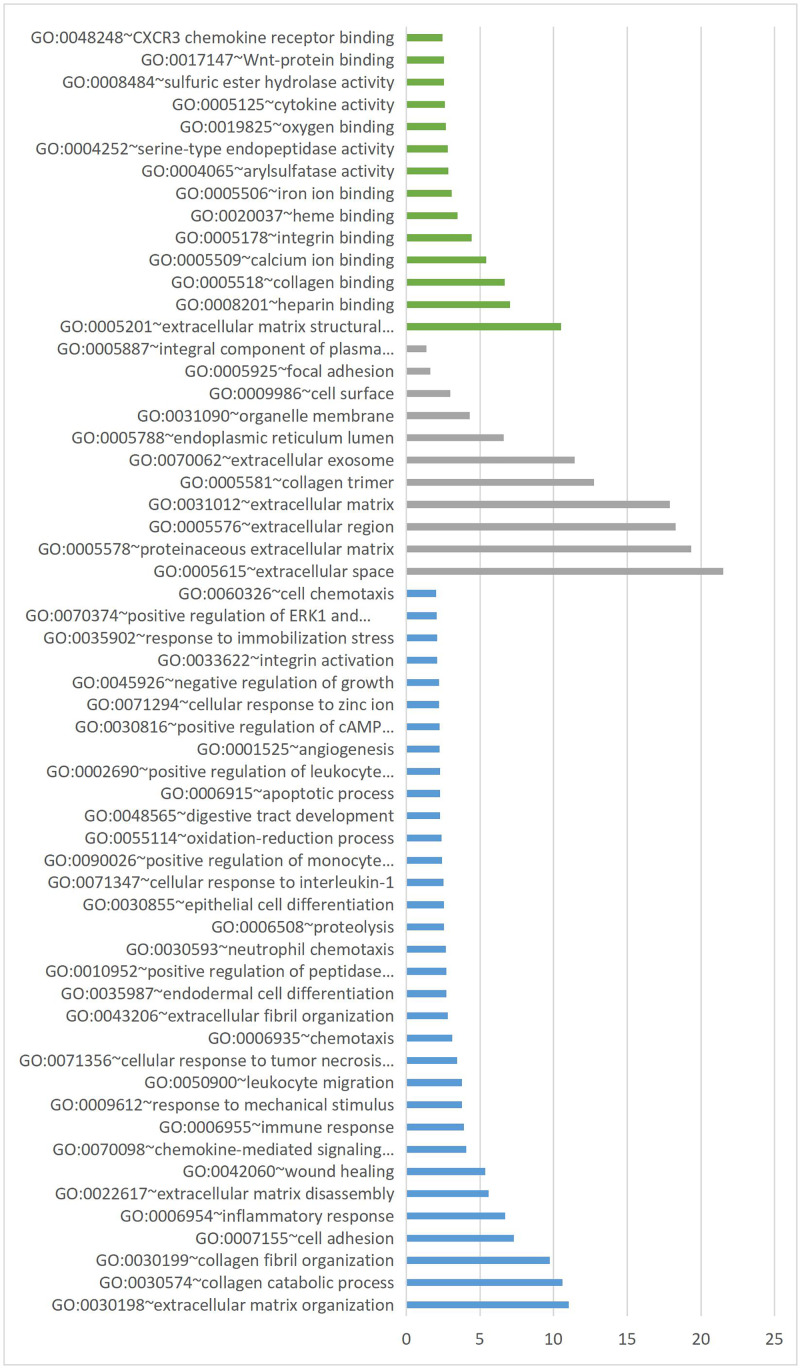
GO enrichment analysis of DEGs on the −log_10_(*P*-value) MF for green bar, CC for gray bar, BP for blue bar.

**Figure 4:**
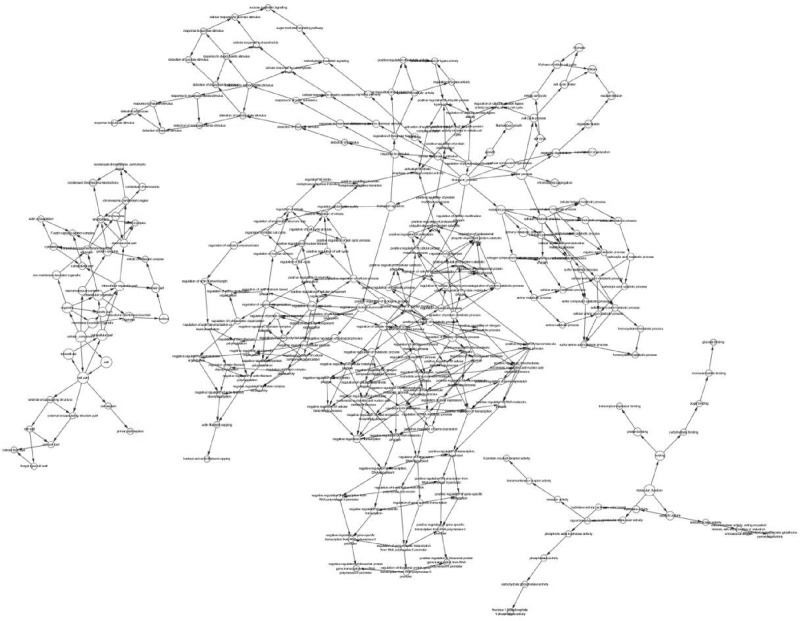
Directed acyclic graph based on the enrichment degree of GO terms Color depth represents the degree of GO terms enrichment.

Using the online analytical tool of DAVID to annotate the DEGs obtained from microarray data integration analysis, the GO function of the DEGs was enriched with a *P*-value <0.05. In GO enrichment analysis, DEGs were divided into three parts: BP, MF, and CC. The statistical results of GO enrichment analysis are shown in Figure 3. In the BP group, DEGs were mainly enriched in ECM formation, collagen synthesis and metabolism, cell adhesion, inflammatory response, and other functions. In the MF group, DEGs were mainly enriched in ECM formation, heparin binding, collagen binding, and other MFs. In the CC group, DEGs were mainly enriched in extracellular region, intracellular space, and exosomes. The GO full analysis is shown in Figure 4.

### KEGG pathway analysis of DEGs

**Figure 5:**
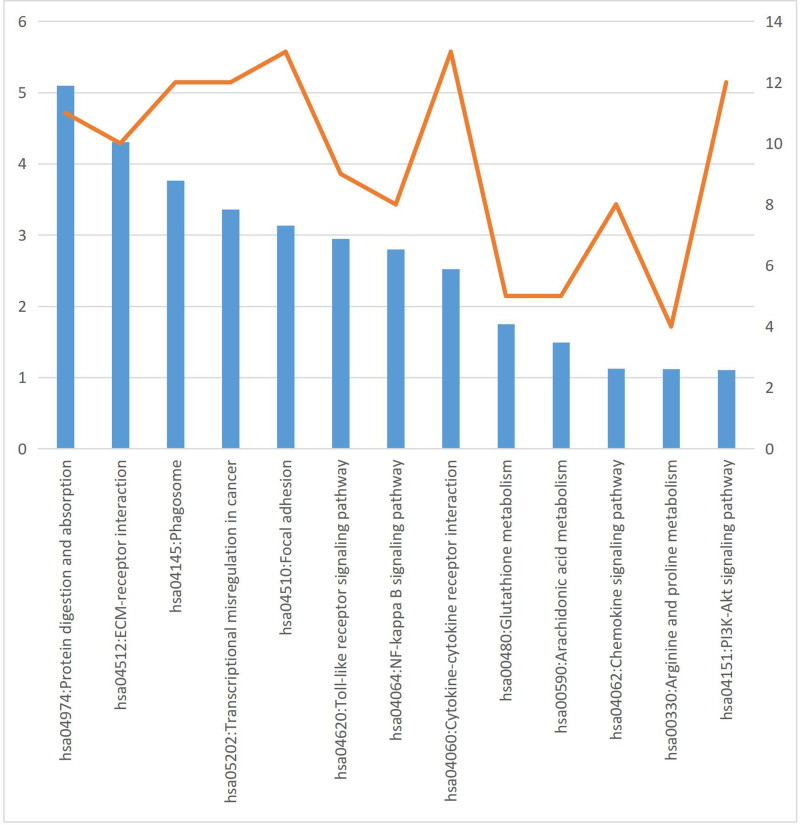
KEGG signal pathway enrichment analysis of DEGs on the −log_10_(*P*-value) (blue bar) and gene count (orange line)

Using the DAVID online analysis tool to analyze the KEGG signal pathway enrichment of the DEGs obtained from microarray data integration analysis, we found that the main enrichment of DEGs was in protein digestion and absorption, ECM receiver interaction, phantom, toll-like receptor (TLR) signaling pathway, focal adhesion, NF-κB signaling pathway, PI3K/Akt signaling pathway, and other signaling pathways. The results are shown in Figure 5.

### PPI network construction and cytoHubba analysis

**Figure 6:**
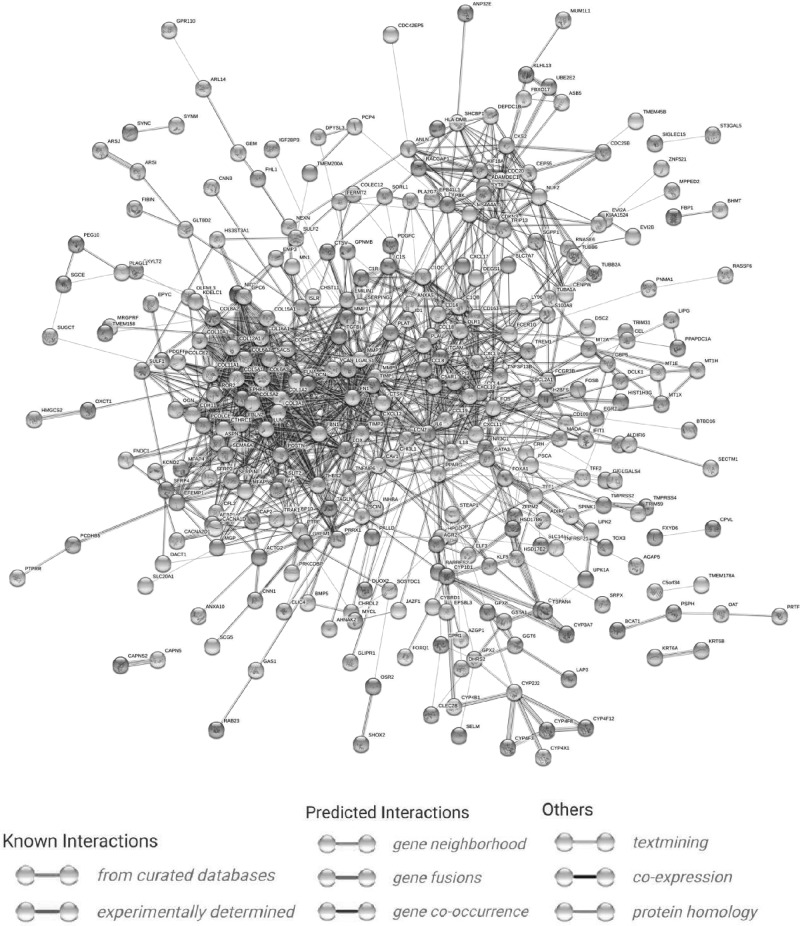
PPI network Circles represent genes, lines represent the interaction of proteins between genes, and the results within the circle represent the structure of proteins. Line color represents evidence of the interaction between the proteins.

**Figure 7:**
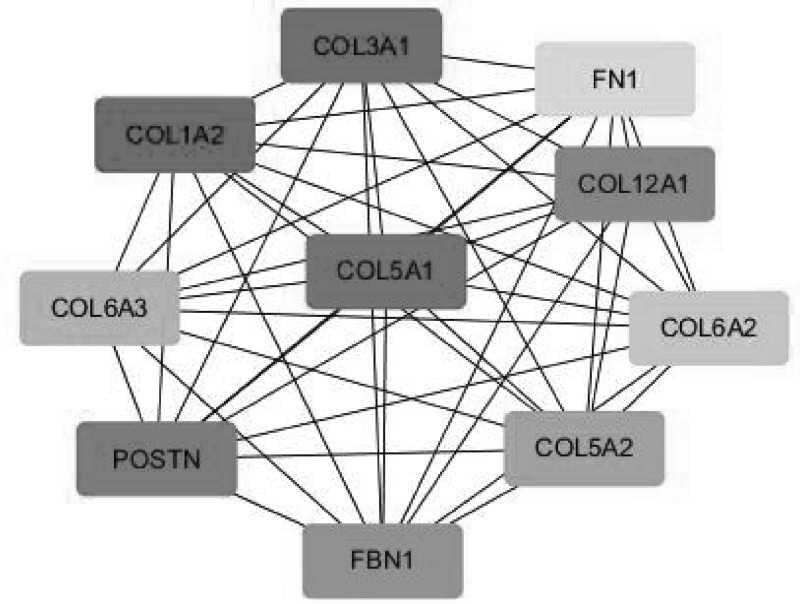
The interaction network of top ten hub genes

The proteins expressed by the DEGs, 389 in total, were analyzed in the PPI network using the STRING database (http://string-db.org), and the PPI network formed by them is shown in Figure 6. The top ten hub genes were screened and ranked (COL1A2, COL3A1, COL5A1, POSTN, COL12a1, FBN1, COL5a2, COL6a3, COL6a2, and FN1) using cytoHubba. The scoring results are shown in Table 2. The interaction network of these hub genes is shown in Figure 7.

**Table 2:** Top ten in PPI network ranked by MCC method

Rank	Name	Score
1	COL1A2	3.08E+08
2	COL3A1	3.08E+08
3	COL5A1	2.96E+08
4	POSTN	2.68E+08
5	COL12A1	2.55E+08
6	FBN1	2.53E+08
7	COL5A2	2.52E+08
8	COL6A3	2.51E+08
9	COL6A2	2.51E+08
10	FN1	2.21E+08

### The Kaplan–Meier plotter

**Figure 8:**
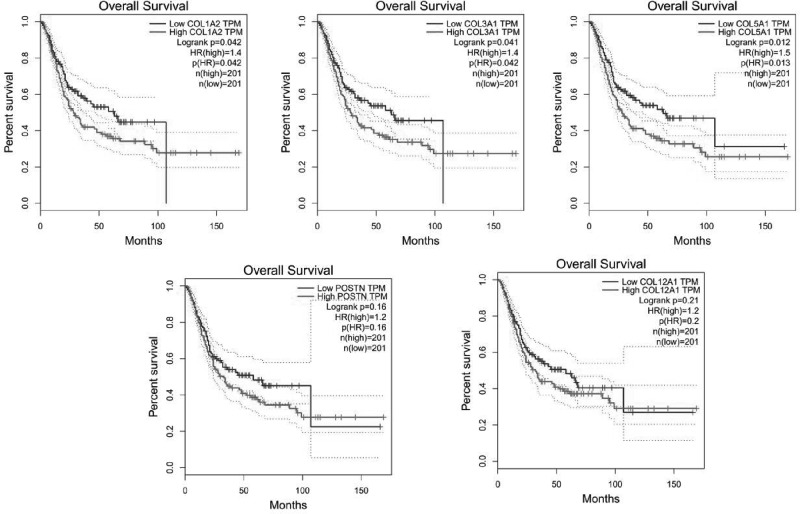
The prognostic value of mRNA level of top five hub genes in bladder cancer patients (OS in Kaplan–Meier plotter)

The Kaplan–Meier curve and log-rank test analyses revealed that the mRNA levels of the top five hub genes (COL1A2, COL3A1, COL5A1, POSTN, and COL12A1) were significantly associated with OS (*P*<0.05) (Figure 8).

## Discussion

Bladder cancer, as a common malignant tumor, can be divided into MIBC and NMIBC depending on whether it invades the muscle layer or not. Treatment of most NMIBC patients can achieve a good therapeutic effect [10]. Once it progresses to the MIBC stage, the specific survival rate of the tumor is 65–78% [11]. Therefore, it is very important to explore the biological mechanism of occurrence and development of MIBC, especially with respect to the development of the disease from the gene level. It is possible that this development may provide us with new ideas and targets for the diagnosis and treatment of MIBC.

Gene chip technology and high-throughput sequencing technology have been widely used in the research of various diseases. These technologies can let us directly describe the expression of thousands of genes. At present, many related articles and data have been published and uploaded to public databases for further research by other scientists.

In the present paper, GSE31684 gene expression profile datasets of bladder cancer were available in the GEO database, and these datasets were analyzed using R software. A total of 389 DEGs were found, including 270 up-regulated and 119 down-regulated genes. After GO enrichment analysis of these genes, it was found that these DEGs were mainly enriched in ECM formation, collagen synthesis and metabolism, cell adhesion, and inflammatory response.The function of epithelial cells, including cell differentiation, migration and invasion, is regulated by interaction with ECM [12], meanwhile collagen is major structural component of the ECM, and tumor development of epithelial cells, including bladder tumors, is often accompanied by ECM changes and remodeling [13]. Recent reports demonstrated that alterations in the ECM microenvironment and collagen synthesis switch likely contributes to bladder cancer progression [14,15].

KEGG signal pathway enrichment analysis showed that the DEGs were mainly enriched in protein digestion and absorption, ECM receiver interaction, phagosome, TLR signaling pathway, focal adhesion, NF-κB signaling pathway, PI3K/Akt signaling pathway, and other signaling pathways.

The PI3K/Akt signaling pathway is an important signaling pathway in the development of many tumors and in inflammation. It has been proved that the PI3K/Akt signaling pathway plays an important role in promoting the proliferation of tumor cells and can regulate cell metabolism, tumor development, migration, and cytoskeleton remodeling, leading to the occurrence, development, and metastasis of tumor cells [16,17]. The activation of PI3K/Akt pathway may also play an important role, including PTEN deficiency and PTEN negative regulation of PI3K level. Inactivation of PTEN associated with p53 has been shown to promote muscle-invasive phenotypes [18]

The NF-κB signaling pathway plays an important role in promoting tumor cell proliferation, vascular production, invasion, and metastasis in the development of bladder cancer, especially bladder urothelial cancer [19–21]. TLRs and the TLR signaling pathway is an important part of the immune system. The TLR-related signaling pathway is involved in the process of epithelial cell proliferation and IgA production. TLRs play an important role in maintaining the tight connection of epithelial cells, recognizing other pathogenic molecular patterns, and inducing the expression of antimicrobial peptides. TLRs can promote inflammation, apoptosis, proliferation, and fibrosis through transduction, acting as tumor promoters [22,23]. The progress of bladder tumor is closely related to the realization of immune escape. The involvement and activation of TLRs and the TLR signaling pathway in bladder tumor can enhance the immune escape of bladder tumor to promote tumor invasion, resistance to apoptosis and resistance to chemotherapy, and finally lead to the progress of tumor [24]. In addition, the activation of TLRs and the TLR signaling pathway in tumor cells can also trigger the activation of the NF-κB signaling pathway, so as to play a role in promoting tumor progression [25].

Next, we used DEGs to build the PPI network (Figure 7) and identify the top ten hub genes: COL1A2, COL3A1, COL5A1, POSTN, COL12A1, FBN1, COL5A2, COL6A3, COL6A2, and FN1. Through these results, we found that the top ten hub genes were all related to the ECM, and seven of them were related to collagen. Collagen is one of the main components of the ECM. At present, there are 19 known collagen subtypes. These proteins are composed of multiple protein chains, which provide tensile strength for tissues and play an important role in the tissue scaffold, cell adhesion, cell migration, cancer, angiogenesis, histo-morphogenesis, and tissue repair [26]. Collagen can promote the metastasis of tumor cells by increasing the effect of epithelial–mesenchymal transition (EMT) [27], which is mainly because of the change in biological stress caused by ECM recombination, resulting in the morphological change of cancer cells, the decrease in adhesion, and the enhancement of metastatic ability [28].

In addition, collagen can promote tumor progression through immune regulation; for example, signaling factors related to neutrophils are significantly increased in the high-density collagen-type breast cancer microenvironment [29]. COL1 can promote the transport of water and macromolecules in tumor cells, and the tumor microenvironment with high-density collagen can promote tumor proliferation by changing tumor metabolism [30,31].

Collagen also has an important relationship with angiogenesis. The inhibition of collagen metabolism has an anti-angiogenic effect. Angiogenesis and survival are closely related to the appropriate collagen synthesized and deposition in the basement membrane [32], especially the structural integrity of COL4, which is the most important for tumor angiogenesis [33]. Periosteal protein (POSTN) is a multifunctional secretory protein, which can be synthesized by both tumor and stromal cells [34]. It plays an important role in the process of tumor EMT [35]. The expression of postn in tumor-related fiber cells or other stromal cells can promote the invasion of pancreatic cancer, ovarian cancer, prostate cancer, esophageal adenocarcinomas, and other malignant tumors [36–38].

## Conclusions

By analyzing the bladder cancer dataset GSE31684 in the GEO database using R software and other tools, a total of 389 DEGs were identified, including 270 up-regulated and 119 down-regulated genes. Through GO function enrichment analysis and KEGG pathway analysis, we found that these genes were enriched in ECM formation, collagen synthesis and metabolism, cell adhesion, inflammation, and other functions, as well as protein digestion and absorption, ECM receiver interaction, phagosome, TLR signaling pathway, focal adhesion, NF-κB signaling pathway, and PI3K/Akt signaling pathway. The PPI network of DEGs was constructed, and the most important ten genes were screened using cytoHubba. Survival analysis of the top five hub genes was carried out using TCGA database data. Our research found that the key genes, including COL1A2, COL3A1, COL5A1, POSTN, and COL12A1, played an important role in the development of MIBC. These results may provide us with a further understanding of the occurrence and development of MIBC, as well as new targets for the diagnosis and treatment of MIBC in the future. However, there were some limitations in the present study that should be declared. The data from GSE31684 have a total of 15 NMIBC samples and 78 MIBC samples, the difference in sample size between the two groups may lead to statistical limitation. Besides, the hub genes on gene expression were only validated in TCGA database and these genes needs to be further validated with more experiment. Future studies will aim to use polymerase chain reaction or Western blotting to verify expression levels of the key genes in samples between MIBC and NMIBC.

## Supplementary Material

Supplementary Table S1

## Data Availability

Data can be obtained from GEO website.

## Abbreviations

BP: biological process
CC: cell component
DAVID: Database for Annotation, Visualization, and Integrated Discovery
DEG: differentially expressed gene
ECM: extracellular matrix
EMT: epithelial–mesenchymal transition
GEO: Gene Expression Omnibus
GO: Gene Ontology
KEGG: Kyoto Encyclopedia of Genes and Genomes
MF: molecular function
MIBC: muscle invasive bladder cancer
NMIBC: non-muscle invasive bladder cancer
PPI: protein–protein interaction
TCGA: The Cancer Genome Atlas
TLR: toll-like recepto
