# Supplementary material for: Extracellular matrix-related genes play an important role in the progression of NMIBC to MIBC: a bioinformatics analysis study
Source: Biosci Rep. 2020 May 26;40(5):BSR20194192. doi: 10.1042/BSR20194192 (PMC7251326; doi:10.1042/BSR20194192)
Supplement: Supplementary Table S1 [file BSR-2019-4192_supp.pdf]

Supplementary Table 1 The clinicopathologic characteristics of the tumor samples

Characteristic	NMIBC(n=15)	MIBC(n=78)
Patient age at RC, median (range), years	69.1(57.4-88.5)	69.3(41.7-91.1)
Gender, number (%)		
Female	4(27%)	21(27%)
Male	11(73%)	57(73%)
Histologic type		
Transitional Cell Carcinoma(TCC)	15(100%)	71(91%)
TCC/Squamous or CIS	0（0）	7(9%)
RC stage, number (%)		
pTa	5(33%)	\
pT1	10(67%)	\
pT2	\	17(22%)
pT3	\	42(54%)
pT4	\	19(24%)
Lymph node status		
pN0	11(74%)	38(49%)
pN+	2(13%)	26(33%)
pNx	2(13%)	14(18%)

RC=Radical cystectomy

CIS=Carcinoma in situ

pNx=Lymph node dissection not done or no lymph nodes analyzed
